# Voice clones sound realistic but not (yet) hyperrealistic

**DOI:** 10.1371/journal.pone.0332692

**Published:** 2025-09-24

**Authors:** Nadine Lavan, Mairi Irvine, Victor Rosi, Carolyn McGettigan

**Affiliations:** 1 Centre for Brain and Behaviour, School of Biological and Behavioural Sciences, Queen Mary University of London, London, United Kingdom; 2 UCL Speech, Hearing and Phonetic Sciences, Division of Psychology and Language Sciences, London, United Kingdom; National Taiwan Normal University, TAIWAN

## Abstract

AI-generated voices are increasingly prevalent in our lives, via virtual assistants, automated customer service, and voice-overs. With increased availability and affordability of AI-generated voices, we need to examine how humans perceive them. Recently, an intriguing effect was reported in AI-generated faces, where such face images were perceived as more human than images of real humans – a “hyperrealism effect.” Here, we tested whether a “hyperrealism effect” also exists for AI-generated voices. We investigated the extent to which AI-generated voices sound real to human listeners, and whether listeners can accurately distinguish between human and AI-generated voices. We also examined perceived social trait characteristics (trustworthiness and dominance) of human and AI-generated voices. We tested these questions using AI-generated voices generated with and without a specific human counterpart (i.e., voice clones, and voices generated from the latent space of a large voice model). We find that voice clones can sound as real as human voices, making it difficult for listeners to distinguish between them. However, we did not observe a hyperrealism effect. Both types of AI-generated voices were evaluated as more dominant than human voices, with some AI-generated voices also being perceived as more trustworthy. These findings raise questions for future research: Can hyperrealistic voices be created with more advanced technology, or is the lack of a hyperrealism effect due to differences between voice and face (image) perception? Our findings also highlight the potential for AI-generated voices to misinform and defraud, alongside opportunities to use realistic AI-generated voices for beneficial purposes.

## Introduction

AI-generated voices are all around us. These synthetic voices are embedded in various technologies and services that we interact with regularly. From giving voices to virtual assistants like Alexa and Siri to automated customer service interactions, and videos requiring voice-overs, it is relatively easy and inexpensive to generate AI-generated voices using a web interface or application programming interface (API). Voice synthesis through AI has already come a long way: Initially, the goal of voice synthesis was to generate intelligible speech that could be understood by listeners. Over time, however, advancements have led to the creation of naturalistic speech that sounds more human-like (for a review see Triantafyllopoulos et al., 2023 [1]). Today, it is possible to engage in naturalistic communication via fully generative AI, where the synthesised voice can at times convincingly mimic vocal identities, intonations, and emotions, making interactions feel more authentic [2].

AI-generated voices can be used to enhance or improve people’s day-to-day experiences. For instance, screen readers equipped with AI-generated voices enable visually impaired users to navigate the internet, read books, and engage with other written content. Similarly, people who have lost their ability to speak can use devices including AI-powered speech synthesis, thus enhancing their opportunities to engage in spoken communication – this can even be personalised via voice cloning to create a better likeness of the original speaker’s voice [3–5]. Other stakeholders also stand to benefit from the ability to artificially generate humanlike speech and voice identities, for example advertisers and social media influencers wishing to generate high rates of speech content while minimising costs (see McGettigan, 2025 [6] for examples). Very recently, the telecommunications company O2 released a voiced AI agent, Daisy, who convincingly emulates a persona designed to waste the time of phone scammers [7]. On the other hand, the same technology that brings these benefits also has the potential for misuse. One of the most concerning applications is the creation of voice clones. These synthetic voices can mimic specific real people with high accuracy, which can be in turn used with malicious intentions [8].

In the context of the potential benefits and risks of AI-generated voices for human societies, it is important to understand how human listeners perceive and evaluate them. For example, just as listeners rapidly form multivariate impressions of people from the sound of their voice alone [9–13], listeners also form (first) impressions based on hearing AI-generated voices. Shiramizu et al. (2002) showed that the fundamental dimensions underpinning the perception of personality traits from AI-generated voices are similar to those found for human voice perception, where, for both types of voices, first impressions or trait judgements are thought to be underpinned by judgements of dominance and valence/trustworthiness. However, while the overall configuration of “voice trait space” may align for human and AI-generated voices, others have reported differences in the evaluation of different kinds of voices. For example, some authors have found that human voices are rated more favourably than AI-generated and other synthetic voices (e.g., higher in likeability, trustworthiness, and/or attractiveness; Bruder et al., 2023 [14]; Cabral et al., 2017 [15]; Kühne et al., 2020 [16]).

The evidence from studies of synthetic or AI-generated voice perception suggests that these voices therefore have the potential to evoke the perception of humanlike traits in listeners, although impressions seem to differ from those generated by human voices. The overall less favourable impressions of AI-generated/synthetic voices have been linked to the lower perceived realness or naturalness of these stimuli [17–19]. With AI-generated voices becoming ever more realistic, it is therefore crucial to establish whether human listeners can successfully distinguish AI-generated voices from authentic recordings of human voices – and what impact this might have on the perception of AI-generated voices more broadly.

Recent studies have addressed the question of how well listeners can distinguish AI-generated voices from human voices, using perceptual classification tasks in which listeners are presented with a range of human and AI/synthetic voice stimuli and are asked to classify them as “real”/ “human” or “fake”/ “AI-generated”. Overall, listeners can achieve classification accuracy rates well above chance across all stimuli. Human voices tend to be more accurately recognised as being human, while AI-generated voices are less accurately recognised as AI-generated [14,20–22]. However, accuracy for labelling AI-generated voices as such (as opposed to as human) can indeed fall to near-chance levels depending on the specific AI-generated identity being heard [22,23]. In sum, this suggests that AI-generated voices are not only increasingly apt to convey humanlike qualities, but are indeed frequently misperceived as human; these observed patterns of classification behaviour and accuracy align with similar findings for the perception of AI-generated faces [24–26].

In the face perception literature, some studies have stepped away from assessing accuracy and have reframed the question to look instead at how often human vs AI-generated images of faces are labelled as “human”. These studies show an AI “hyperrealism” effect, where AI-generated face images are more often judged to be human [27–29], and are rated as more realistic [30], than images of real human faces. Given ongoing and rapid advancements in the sophistication of AI-generated voices, we can therefore now also ask whether state-of-the-art generative AI for voices has also reached a state of hyperrealism, and what the broader perceptual properties of state-of-the-art AI-generated voices are.

This question of realness in speech audio is not only of interest for the application of AI voice technology in real-world scenarios. It is also relevant to theories of voice perception by humans, which have to date considered only human voices and not artificial voices (e.g., [11,31,32]. A recent theoretical proposal considers how voice naturalness (a concept closely aligned with what we term “realness”) is processed in relation to synthetic voices (Nussbaum et al., 2025 [33]). Informed by the existing literature, the authors’ proposed framework conceptualises the perceived naturalness (or realness) of voice/speech audio via its relative deviance from a (prototypical) human voice as the maximally natural (or real) reference. Our study crucially contributes to this research area both on an empirical and theoretical level: By asking about whether there are voices that are *more* natural or real than human voices, we extend current proposals of naturalness and realness perception to consider voices that sound *more* natural or real, relative to (a prototype of a) human voice. This in turn raises questions about whether the most human-sounding voices indeed need to have been produced by a human, thus further calling for careful delineations of objective characteristics of a voice (e.g., this voice is real because it was produced by a human) vs subjective perceptual characteristics of a voice (e.g., this voice sounds real, even though it was AI-generated; [33]).

## Experiment 1a and 1b

In our first experiment, we examined whether a hyperrealism effect can be observed for AI-generated voices. Given that there are several avenues through which synthetic voices can be generated that differ substantially from one another, we opted to include two different types of AI-generated voices, using current state-of-the-art voice synthesis tools: We, first, created AI-generated voices from within the latent space of ElevenLabs’ “Voice Design” model (Experiment 1a, labelled as “generic AI-generated voices” in this study). Generic AI-generated voices of this particular style are currently used to generate novel vocal identities, and used for example as voice-overs for advertisements and online content videos, or to narrate audiobooks or podcasts. Second, we also generated voice clones, by using recording from existing voices to create a ‘clone’ of each. These voices are intended to closely mimic the particular voice that they have been cloned from (Experiment 1b, labelled as “voice clones” in this study).

We then compared perceptual judgements of how real or human these generic AI-generated voices and voice clones sounded compared to recordings of real human voices. If a hyperrealism effect exists for voices, AI-generated voices would be perceived as more real-sounding than human voices. We predicted that this hyperrealism effect would most likely be observed for cloned voices, given their intention to emulate a specific human voice identity, thus likely sounding maximally human. We tentatively predicted that a hyperrealism effect may also be present for other (non-clone) generic AI-generated voices. To investigate broader perceptual consequences of how generic AI-generated voices are perceived in relation to human voices, we also collected judgements of how trustworthy and dominant the different voices sounded, as these two traits respectively load on the orthogonal valence and dominance dimensions of voice trait space characterised in previous work (e.g., [12,34,35]. Based on the previous literature that suggests that AI-generated voices are perceived less positively on the valence dimension (e.g., less likeable, less attractive; [14–16]), we predicted that AI-generated voices in our study should be perceived as less trustworthy than human voices. For dominance, we predicted that there may be an effect of voice type but made no directional predictions, as dominance is conceptualised to be orthogonal to valence percepts and given no prior findings in relation to this dimension for AI-generated voice perception [12,35].

## Methods

### Participants

50 participants each took part in Experiments 1a and 1b. All participants were recruited from Prolific.co, were aged between 18 and 65 years old, had no self-reported hearing difficulties, and were all native speakers of English, resident in the UK. In Experiment 1a, 29 of the 50 participants were female, 21 were male, and the mean age of the sample was 36.4 years (SD = 10.6 years). In Experiment 1a, 22 of the 50 participants were female, 28 were male, and the mean age of the sample was 36.8 years (SD = 10.2 years). All participants were paid at a rate of £10 per hour. The study was approved by the local ethics chair for Speech, Hearing, and Phonetics Sciences as part of the UCL Research Ethics Committee (approval number: SHaPS-2023-CM-038, approval granted on the 27^th^ of July 2023). All data for Experiments 1a and 1b were collected on the 8^th^ of October 2024.

All participants passed all the vigilance trials included in the task (see Procedure), such that we made no exclusions.

### Materials

Experiment 1a and 1b both included 80 voices in total, comprising 40 human voices and 40 AI-generated voices. Each voice was represented by one stimulus recording, in which the (Human or AI-generated) voice produced one of four sentences from the Rainbow Passage ( [36]; e.g., “*The rainbow is a division of white light into many beautiful colours”)*. By including full sentences as stimulus materials, our definition of “voice perception” in the context of human and AI-generated voices thus both entails paralinguistic cues, such as the quality of the voices, and linguistic features, such as pronunciation of certain phonemes.

The same human voices and stimuli were used in Experiment 1a and 1b: These 40 voices were selected from the CSTR VCTK database [37], a database of speech recordings from 110 speakers of English. Speaker selection was restricted to accents that are offered by the voice synthesis tool we used, i.e., ElevenLabs’ Voice Design model (https://elevenlabs.io). As a result, we included British, American, Australian, and Indian accents (note that, at the time of stimulus generation, ElevenLabs’ Voice Design also offered “African” but this accent label was too general for our purposes). When selecting British-accented voices from the database, we restricted our inclusion to speakers from the South/South-East of England including London, and to any other British voices that spoke perceptible with a standard Southern British English Accent. One speaker labelled as Indian in the database spoke with a North American accent and thus was included as American in our study. The final set of 40 human voices included 18 American speakers (15 female, 3 male), 18 British (11 female, 7 male), 2 Australian (both male), and 2 Indian (1 female, 1 male). We only considered including recordings from these speakers in which they did not make any obvious reading mistakes (e.g., replacing/skipping words, correcting errors).

We used two types of AI-generated voices in Experiment 1a and 1b: For Experiment 1a, we created 40 AI-generated voices using ElevenLabs’ Voice Design function, which at the time of stimulus generation created synthetic voices for a specific gender (male vs female), age (young vs. middle-aged vs. old), and accent (e.g., “British”, “American”, “Indian”). Each synthetic voice was matched in gender and accent to one of the 40 selected human voices. All human voices were aged between 18–33 years (mean age: 23 years), so we used the “young” setting in ElevenLabs’ “Voice Design” for all voices. We furthermore used the default setting for “Accent strength” in ElevenLabs. For these 40 voices, we first generated a test sentence to check for broad perceptual matches for the desired demographic (e.g., does the synthesised voice indeed sound male and British-accented?). If this was not the case, we asked ElevenLabs to generate a new voice until a perceptible accent and gender match was achieved. Once a match was achieved, we generated 1 of the 4 Rainbow Passage sentences also used in the human speaker set to create the stimuli for synthetic voices used in the task. We re-generated any stimuli that included perceptible audio artefacts which were overlapping with the speech.

For Experiment 1b, we created 40 voice clones of the 40 human voices from the VCTK data set. We used ElevenLabs’ Instant Voice Cloning function (with the Eleven Multilingual v2 model) to do this. Cloned voice identities were generated based on around 4 minutes of recordings of each speaker. The data comprised a concatenation of recordings of read sentences from the VCTK corpus. Sentences did not necessarily overlap between the different voices. We then generated one of the four Rainbow Passage sentences per voice clone, which was checked for obvious audio artefacts overlapping with the speech signal and regenerated where necessary. Original human voices and their respective clones were never represented by the same sentence in the final stimulus set.

We further pre-processed all recordings by removing background noise and artefacts using *rnnoise* (https://github.com/xiph/rnnoise), trimmed silences using a Python interface of Google’s voice activity detector (VAD; *webrtcvad)* (https://github.com/wiseman/py-webrtcvad), and removed breaths and other noises using *librosa* [38]*.* The results of this processing pipeline were manually checked. Finally, the final versions of the stimuli were RMS normalised for sound level using *ffmpeg-normalize* (https://github.com/slhck/ffmpeg-normalize) and resampled to 22050 Hz.

We note that this extensive pre-processing procedure was applied to create ‘ideal’ human and AI-generated sentences that were free of obvious artefacts (be they human- or AI-generated). This was done in line with the approach of recent studies investigating (hyper)realism in the perception of AI-generated faces (Nightingale & Farid, 2022; Miller et al., 2023), whose authors reasoned that screening of AI-generated stimuli to avoid obvious rendering errors is representative of how real-world users would screen images (e.g., to intentionally deceive human perceivers). By extension of this logic, and so as not to bias our stimulus set by only heavily preprocessing the AI-generated stimuli, we also used a set of ‘ideal’ human recordings (without breaths, clicks, stutters, hesitations).

In sum, the term “AI-generated” when applied to our stimuli refers to the use of artificial intelligence to learn and generate the characteristics of human speech and voice audio within ElevenLabs’ Voice Design and Instant Voice Cloning tools. However, stimulus creation was not fully generative: Human (experimenter) intervention was used to nominate the target gender, age, and accent parameters within the Voice Design tool, to specify the text for generating the required text-to-speech output in both the Voice Design and Instant Voice Cloning models, and to apply appropriate screening for inaccuracies and artefacts (with regeneration or editing applied as needed).

### Procedure

The experiment was run using Gorilla Experiment Builder [39]. Participants first read an information sheet and then provided informed consent. Participants then proceeded to complete two rating tasks, in which they rated all 80 voices for trustworthiness or dominance (“How trustworthy/dominant does this voice sound?” 0 - “Not at all”, 100 - “Extremely”). Trust and dominance were rated in separate tasks, with their order counterbalanced across participants. After these tasks had been completed, participants were informed that some of the voices in the experiment were recordings of real humans, and others were AI-generated audio in order to contextualise the following two tasks. Participants then moved on to rate the perceived ‘realness’ of the voices (“How real does this voice sound?” 0 – “Not at all”, 100 – “Extremely”). For all three ratings tasks (dominance, trustworthiness, realness), participants registered their response on a continuous slider scale. As a final task, participants judged whether a voice recording had been produced by a real human or was AI-generated audio, in a binary forced choice task (labelled as the “Human or AI-generated” classification task in this paper). Participants registered their response for this task via a mouse click. The order of stimuli was randomised for each task. We added the realness rating to directly assess perceived realness in continuous manner, as opposed to solely using the less direct, categorical human vs AI judgements reported in previous research to assess “realism” or “naturalness” of voices (e.g., Barrington & Farid, 2024; Mai et al., 2023; Müller et al., 2024).

To determine the specific nature of our “realness” scale, we ran a pilot study: We asked 15 participants to use the “realness” scale (0 – not real at all, 100 – extremely real), while another 13 participants were asked to use the bipolar scale ranging from 0 indicating “sounds AI-generated” to 100 indicating “sounds human”. All participants then completed the binary “AI-generated” vs “Human” judgement, which was also included in the final experiment. We found high correlations of mean ratings per sound file between three measures (two scales and the binary forced choice task, rs > .7). Furthermore, interrater agreement, as measured through the intra-class correlation coefficient (ICC(2,k)) was very high for the two rating scales (> 0.79). We interpreted the high correlations and high inter-rater agreement as evidence that, despite using different labels and different types of response options, participants tapped into similar concepts for all 3 measures. As a result, we considered the specific labels and tasks to be largely interchangeable for the purposes of our study.

In all three ratings tasks and the “Human or AI-generated” classification task, participants completed 5 vigilance trials per task in which a voice recording instructed them to give a specific response (e.g., “Please select 7 on the next screen”).

After completing all tasks, participants completed debrief questions to check that they experienced no technical difficulties, alongside questions examining how participants tried to identify whether a voice was human or AI-generated. The experiment took on average around 40 minutes to complete.

We calculated the intra-class-correlation coefficient (ICC(2,k)) for all three rating scales to assess data quality and found that ICCs were high (> 0.73) throughout for both Experiments 1a and 1b.

### Data, code, and materials availability, open science practices

The data, stimuli, and analysis code for all experiments have been shared on the Open Science Framework: https://osf.io/mrw5q/. Neither experiment was pre-registered.

## Results and discussion

### Is there a hyperrealism effect for voices?

To assess whether there is a hyperrealism effect for AI-generated voices, we compared realness ratings and binary humanness judgements (from the “Human or AI-generated” classification task) for human vs AI-generated voices and human voices vs voice clones (Experiments 1a and 1b, respectively).

**Fig 1:**
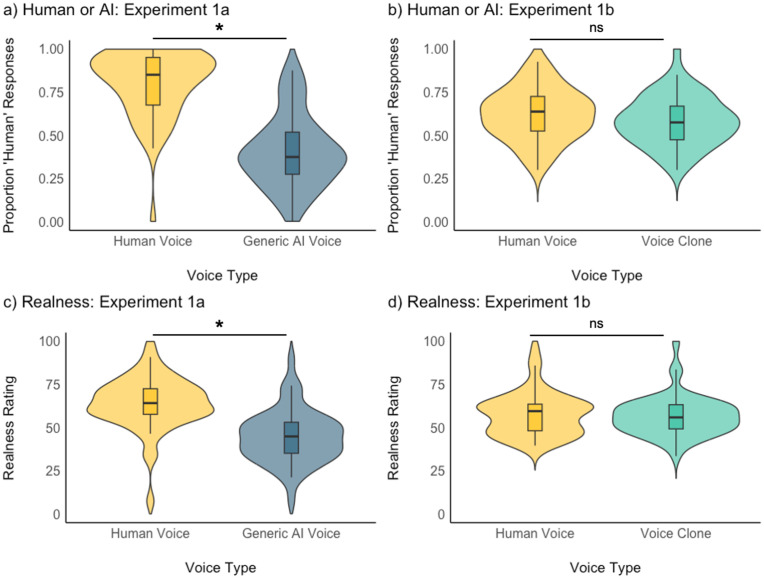
Violin plots showing the results for the forced choice “Human or AI” classification task and Realness rating task for Experiment 1a (panel a, c) and Experiment 1b (panel b, d). Violin plots show the distribution of the data with boxplots. * indicates p < .05 for the effect of Voice Type in a (G)LMM.

Following Miller et al. (2023), we analysed the “Human or AI-generated” classification task in a generalised linear mixed model (GLMM) using the *lme4* package [40] in *R.* Voice Type (Human Voice vs Generic AI-generated Voice/Voice Clone) was entered as a fixed effect and participant and stimulus as random intercepts. We did not model random slopes to avoid overfitting our data, after encountering convergence issues. We found a significant effect of Voice Type (OR = 0.12, CI = 0.08-0.16; p < .001) in Experiment 1a, with Generic AI-generated Voices being labelled as “Human” significantly less often than Human Voices (Mean_Generic AI-generated Voices_ = 0.41, Mean_Human Voices_ = 0.81, see Fig 1a). For Experiment 1b, there was, however, no significant difference between voice types (OR = 0.84, CI = 0.62-1.13; p = .239, Fig 1b), such that Voice Clones and Human Voices were labelled as “Human” similarly often (Mean_Voice Clones_ = 0.58, Mean_Human Voice_ = 0.62).

A similar picture emerged for the realness ratings. LMMs on realness ratings with Voice Type (Human Voice vs Generic AI-generated Voice/Voice Clone) as a fixed effect and participant and stimulus as random intercepts showed a significant effect of Voice Type in Experiment 1a (ß = −19.29, CI = 22.89-15.70; p < .001, Fig 1c). As for the Human vs AI-generated task, Generic AI-generated Voices were rated as sounding less real than Human Voices (Mean _Generic AI-generated Voices_ = 44.7, Mean_Human Voices_ = 64.0). There was no effect of Voice Type in Experiment 1b (ß = −1.05, CI = −4.65-2.55; p = .568, Fig 1d), such that Voice Clones and Human Voices sounded similarly real (Mean_Voice Clones_ = 56.6, Mean_Human Voices_ = 57.6).

We therefore find no evidence in either of our two tasks or Experiment for an overall hyperrealism effect in AI-generated voices. Generic AI-generated Voices are perceived as less real/human than Human Voices, while Voice Clones and Human Voices are consistently perceived to be similarly real/human.

Strikingly, on average, for 41% of Generic AI-generated Voices trials and 58% of Voice Clones trials, listeners labelled these AI-generated voices as being human in the “Human or AI-generated” classification task. These data thus underline how convincing Generic AI-generated Voices can already be, with participants often mistaking Generic AI-generated Voices as real human voices. In light of this observation, we next analysed how well listeners were able to tell Generic AI-generated Voices from human voices, while also considering response biases.

For an analysis of how accent (American vs British English) affects voice perception, please see S1 File. In brief, our participants were from the UK in all experiments, and perhaps as a result of familiarity with the accent, voices with British English accents were rated as more real and as more likely to be human than voices with American accents. Accent did not interact with voice type in a systematic manner.

### How good are people at judging whether a voice is AI-generated or human?

To assess performance on how good listeners are at judging whether voices are AI-generated or human, we ran a signal detection analysis. To this end, we calculated D Prime and Criterion C for the “Human or AI-generated” classification task for Experiments 1a and 1b, respectively. Hits were defined as participants listening to human voices and correctly judging them as human voices, and false alarms were defined as participants listening to AI-generated voices and identifying them as human voices. D Prime is a measure of discriminability, with 0 indicating that participants were not able to distinguish human from AI-generated voices, that is, they had no sensitivity to identify the signal (here: the human voice). Increasingly positive D Prime tracks increasing sensitivity to being able to detect human voices (while rejecting AI-generated voices as human voices). C is a measure of bias, with 0 indicating that no bias is present, negative values for C indicating a bias towards responding “Human” and positive values for C indicating a bias towards responding “AI-generated”.

**Fig 2:**
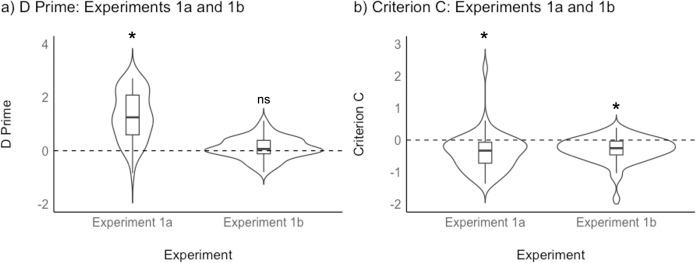
Violin plots showing the results for sensitivity analyses conducted on the “Human or AI” classification task. Panel a) shows D Prime, Panel b) shows Criterion C for Experiment 1a and 1b. Violin plots show the distribution of the data with boxplots. * indicates p < .05 in a one-sample t-test against 0.

Using one-sample t-tests against 0, we found that D Prime for Experiment 1a was significantly above 0 (M = 1.30, SD = 0.91, t(49) = 10.13, p < .001, Cohen’s *d = 1.42;* Fig 2a). Listeners were therefore able to distinguish between Human Voices and Generic AI-generated Voices with some degree of sensitivity. In Experiment 1b, D Prime was, however, not significantly above 0 (M = 0.10, SD = 0.41, t(49) = 1.68, p = .100, Cohen’s *d = 0.24*), such that listeners had no sensitivity to distinguish Human Voices and Voice Clones.

C was significantly lower than 0 for both Experiment 1a (M = −0.38, SD = 0.60, t(49) = −4.47, p < .001, Cohen’s *d = 0.63;* Fig 2b) and Experiment 1b (M = −0.29, SD = 0.40, t(49) = −5.18, p < .001 Cohen’s *d = 0.73*), such that listeners were biased towards judging the voices included in the experiments as human.

These findings complement the analysis of the raw responses for the Human vs AI task reported above, showing that, when accounting for biases, listeners cannot tell the difference between human voices and voice clones, while they can distinguish between human and generic AI-generated voices.

### Do AI-generated voices and voice clones differ from human voices in their perceived social traits?

**Fig 3:**
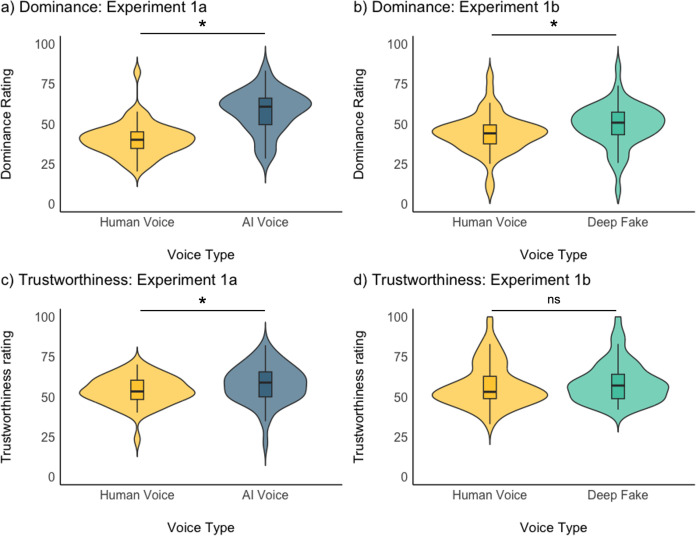
Violin plots showing the results for the trait ratings tasks for Experiment 1a (panels a, c) and Experiment 1b (panels b, d). Violin plots show the distribution of the data with boxplots. * indicates p < .05 for the effect of Voice Type in a (G)LMM.

We next asked whether and how AI-generated voices are perceived differently from human voices in terms of core social traits (dominance and trustworthiness, see McAleer et al., 2014; Oosterhof & Todorov, 2008) [12,35]. Linear mixed models for each trait and experiment, with Voice Type as a fixed effect and participants and stimulus as random intercepts, showed that AI-generated voices were perceived as significantly more dominant than human voices in both Experiment 1a (Mean_Generic AI-generated Voice_ = 58.1, Mean_Human Voice_ = 40.4; ß = 17.67, CI = 13.79-21.55; p < .001, Fig 3a) and Experiment 1b (Mean_Voice Clone_ = 49.4, Mean_Human Voice_ = 44.0; ß = 5.48, CI = 1.07-9.90; p = .015, Fig 3b).

For trustworthiness, generic AI-generated Voices in Experiment 1a were perceived as significantly more trustworthy than Human Voices (Mean_Generic AI-generated Voice_ = 57.9, Mean_Human Voice_ = 53.8; ß = 4.15, CI = 1.49-6.80; p = .002, Fig 3c). Voice Clones were rated higher in trustworthiness than Human Voices, although this difference was not significant (Mean_Voice Clone_ = 58.9, Mean_Human Voice_ = 57.1; ß = 1.86, CI = −0.57-4.30; p = .133, Fig 3d).

As has been reported in previous studies [14,16,19], there seem to be some differences in how human vs AI-generated voices are evaluated. However, while previous studies have reported that AI-generated voices are evaluated less positively particularly when perceived as less natural, we now see some evidence for higher trustworthiness evaluations and clear evidence for higher dominance ratings. This finding aligns with other recent work in which we have reported higher ratings on the warmth and competence dimensions of social trait space for voice clones compared with human voice recordings, but only when listeners are unfamiliar with the human identities .– For familiar voice identites (be they other familiar voices or the self voice), favourable trait ratings are dependent on the perceived similarity of the voice clone to the speaker’s real voice [23,41].

There are some aspects of the data for Experiments 1a and 1b that warrant further investigation. For example, we observe sizeable differences in the assessment of human voices: The human voice stimuli used in Experiment 1a and 1b were identical, as were the tasks used. The only change in the experimental design was in terms of the type of AI-generated voices we included. Nonetheless, evaluations for how human/real the human voices sounded in Experiment 1a and Experiment 1b differ substantially, particularly in the “Human or AI-generated” classification task, with listeners labelling 81% vs 62% of the human voices as “human”, respectively. Given that the Hyperrealism effect is assessed via a relative difference in perceived “realness” between human voices (which form a baseline here) and AI-generated voices, these context effects somewhat complicate a synthesis of the results across the two experiments. We therefore amended our experimental design for Experiment 2, which aimed to replicate the key findings of both Experiment 1a and 1b.

## Experiment 2

We found no evidence for a hyperrealism effect in Experiment 1a and 1b. However, we observed that our “baseline” condition, the perceived realness/humanness of human voices, was substantially affected by the broader context in which these human voice recordings were presented. That is, in the context of the generic AI-generated voices in Experiment 1a, which sounded substantially less real than the human voices, the human voices were rated as very real. Intriguingly, the same human voices were rated as substantially less real or human-sounding in Experiment 1b, when compared to Voice Clones. This potentially reflects that the perceived realness of human voices is amplified in the context of voices that sound more clearly AI-generated. At the same time, the lower realness/humanness judgements in Experiment 1b may be the result of participants second-guessing their judgements in the face of overall more real- and human-sounding Voice Clones.

This observation of realness and humanness judgements being influenced by the broader stimulus context is also pertinent in relation to the reported hyperrealism effect in the face perception literature (e.g., Miller et al., 2023). Here images of human faces were only labelled as human around 50% of the time. For a similar task using AI-generated voices, Barrington et al. (2025) [20] found only 67% of human voices were labelled as “human” in a task including voice clones and real human voices. The surprisingly low proportion of “human” responses to human stimuli in the context of very realistic (and, sometimes, hyperrealistic) AI-generated faces and voices in other studies is similar to the lower realness ratings for human voices in the context of the more realistic voice clones in our Experiment 1b (compared to Experiment 1a).

In Experiment 2, we therefore aimed to replicate our findings from both Experiment 1a and 1b while eliminating these context effects. We achieved this by including all types of voices (Human Voices, Generic AI-generated voices, Voice Clones) in the same experiment, such that they were presented in a single unified context, which makes relative comparisons across the two types of AI-generated voices more interpretable.

We expected to replicate the pattern of results observed in Experiment 1a and 1b, where AI-generated voices were perceived to be less real and less human-sounding than human voices, while voice clones were perceived to be similarly real and human.

## Methods

### Participants

50 participants took part in Experiment 2. The selection criteria for participants were identical to Experiment 1a and 1b. In Experiment 2, 30 of the 50 participants were female, 20 were male, and the mean age for this sample was 38.7 years (SD = 10.2 years). All participants were again paid at a rate of £10 per hour. The study was approved by the same ethics committee as Experiments 1a and 1b. All data for Experiment 2 were collected on the 14^th^ of October 2024.

All participants passed the vigilance trials included in the task, such that no exclusions were made.

### Materials

The stimuli in Experiment 2 were the same as in Experiment 1a and 1b: For Experiment 2, we combined the stimuli from the two previous experiments to create a stimulus set of 120 voices (40 Human Voices, 40 AI-generated Voice Clones, 40 Generic AI-generated voices).

### Procedure

The procedure of Experiment 2 was identical to that of Experiments 1a and 1b with the exception that we no longer included the trustworthiness and dominance ratings tasks. Due to combining all stimuli from Experiments 1a and 1b, participants now rated 120 stimuli per task instead of 80 stimuli as was the case in Experiments 1a and 1b. The experiment took on average around 25 minutes to complete.

## Results and discussion

As in Experiments 1a and 1b, agreement between raters on the realness scale was very high (ICC(2,k) = 0.89).

We again analysed the Human vs AI classification task and the realness ratings to determine whether the trends we had observed across Experiments 1a and 1b (Human Voices = Voice Clone > Generic AI-generated voice) could be replicated when including both types of AI-generated voices within the same task. We again ran a GLMM for the Human vs AI judgements task and an LMM for the realness ratings with Voice Type (Human Voice, Generic AI-generated Voice, Voice Clone) as a fixed effect and participant and stimulus as random intercepts.

**Fig 4:**
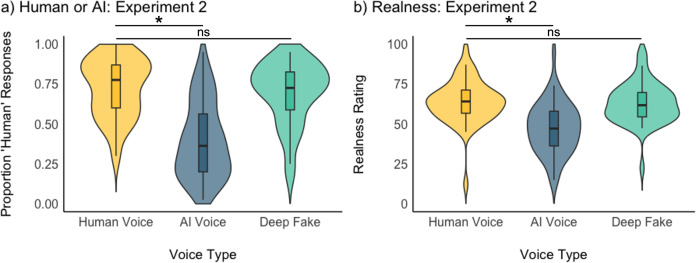
Violin plots showing the results for the forced choice Human or AI classification task (panel a) and Realness rating task (panel b) for Experiment 2. Violin plots show the distribution of the data with boxplots. * indicates p < .05 for the effect of Voice Type (relative to “Human Voice” as a reference condition) in a (G)LMM.

There were significant effects of Voice Type for the “Human or AI-generated” classification task and the Realness ratings. Specifically, in the binary choice task, Generic AI-generated voices were labelled has "human" significantly less frequently than human voices (Mean_Generic AI-generated Voice_: 0.39, Mean_Human Voice_: 0.72; OR = 0.18, CI = 0.14-0.25; p < .001, Fig 4a), while Voice Clones were labelled as "human" with similar frequency to Human Voices (Mean_Voice Clone_: 0.70, Mean_Human Voice_: 0.72; OR = 0.86, CI = 0.63-1.17; p = .341, Fig 4a). The same pattern emerges for Realness ratings, where Generic AI-generated voices were perceived as significantly less real than Human Voices (Mean_Generic AI-generated Voice:_ 47.4, Mean_Human Voice_: 63.8; ß = −16.42, CI = −20.44-12.40; p < .001, Fig 4b), while Voice Clones were perceived to be similarly real to Human Voices (Mean_Voice Clone_: 63.0, Mean_Human Voice_: 63.8; ß = −0.84, CI = −4.86-3.18; p = .682, Fig 4b).

Interestingly, the realness ratings and humanness judgements in Experiment 2 for Human Voices fall between the ratings of Human Voices observed in Experiment 1a and 1b. Similarly, the perceived realness and humanness for Generic AI-generated Voices remain broadly the same, while Voice Clones are also perceived as more real and more human than in Experiment 1b. This may suggest that the context effects observed in Experiment 1 arise from listeners both boosting the evaluations of how real Human Voices (and Voice Clones) sound in the context of less convincingly real-sounded Generic AI-generated voices (Experiment 1a), while also second-guessing their responses in context of very convincing Voice Clones (Experiment 1b).

**Fig 5:**
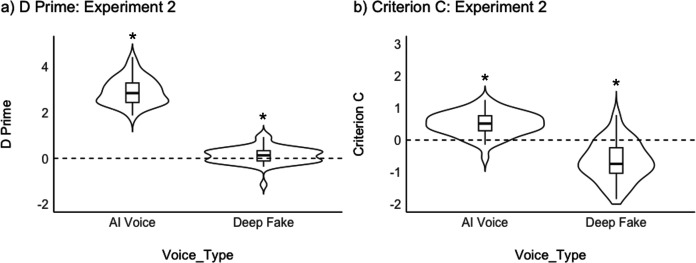
Violin plots showing the results for sensitivity analyses conducted on the forced choice Human or AI judgements task. Panel a) shows D Prime, Panel b) shows Criterion C for Experiment 2. Violin plots show the distribution of the data with boxplots. * indicates p < .05 in a one-sample t-test against 0.

A sensitivity analysis of D Prime and Criterion C largely replicated our findings from Experiments 1a and 1b. For Experiment 2, we this timel calculated two separate D Prime and C scores for each participant: one to assess the sensitivity of distinguishing Generic AI-generated voices from Human Voices, the other to assess the sensitivity of distinguishing Voice Clones from Human Voices. D Prime calculated for AI-generated voices was significantly above 0 (Mean = 2.93, SD = 0.58; t(49) = 35.71, p < .001, Cohen’s *d = *5.06), while D Prime for distinguishing Voice Clones from human voices was much lower (Mean = 0.13, SD = 0.36; t(49) = 2.44, p = .018, Cohen’s *d = *0.33; Fig 5a). In contrast to Experiment 1b, the latter t-test was significant, although mean D Prime is still very low.

Criterion C calculated for distinguishing Generic AI-generated Voices and Human Voices was significantly above 0 (Mean = 0.51, SD = 0.36; t(49) = 9.86, p < .001, Cohen’s *d = *1.42; Fig 5b), indicating a small bias to label voices as AI. This is the opposite to what we found in Experiment 1a and may be linked to the context effects observed in Experiments 1a and 1b. For example, with more voices (Human Voices and Voice Clones) sounding convincingly real, Generic AI-generated Voices were now put into a context in which they sounded the least real out of all of the stimuli. This perceptual pattern may underpin this bias. Criterion C for distinguishing Voice Clones from Human Voices was significantly below 0 (Mean = −0.65, SD = 0.60; t(49) = −7.67, p < .001, Cohen’s *d = *1.08), suggesting a bias to respond “human”, as observed in Experiment 1b.

### Hyperrealistic voice clones for individual voices?

**Fig 6:**
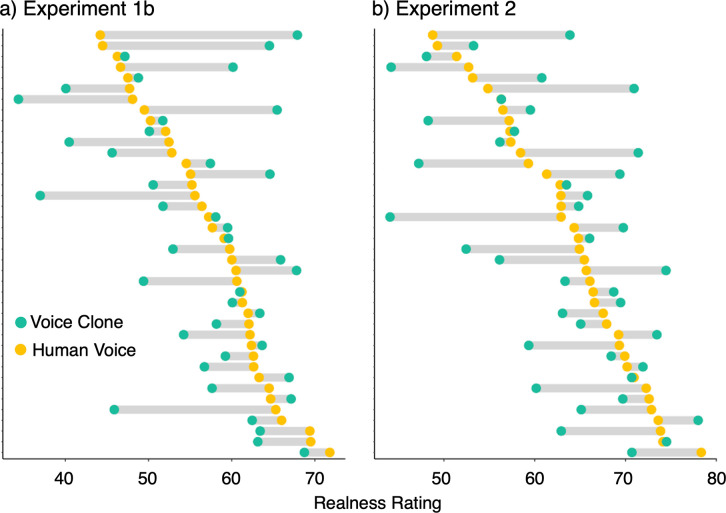
Mean realness ratings for the Human Voices (yellow dots) and their corresponding Voice Clones (turquoise dots) from Experiments 1b and 2. Grey bars illustrate the difference in ratings between a Human Voice and its corresponding Voice Clone.

There is no evidence for a hyperrealism effect at a group level. However, with a view towards effects at the level of individual voices, we below plot mean realness ratings per Human Voice and its corresponding Voice Clone (Fig 6). We do not consider the Generic AI-generated voices included in Experiments 1a and 2 here, as they have no direct counterparts among the human recordings.

While this is not a formal analysis and is only based on a single short stimulus, this plot illustrates that these are item-based differences in the perceived realness of human recordings and voice clones. When relating realness ratings for human voices and their corresponding voice clones, we can then also see that for some human-voice clone pairs, the Voice Clone sounds much less real, while for others there is a ‘hyperrealism’ effect. This plot therefore raises questions about what drives these differences in perceived realness, both in the human recordings as well as the AI-generated voice clones.

## General discussion

We show that, under certain conditions, it is not possible for human listeners to accurately discriminate between AI-generated voices and genuine recordings of human voices. Although we did not find evidence of a hyperrealism effect at a group level – as AI-generated voices were not overall labelled as “human” more often and/or rated higher in realness compared with human voices – we find that, in the particular case of voice clones, listeners are consistently biased towards labelling these AI-generated voices as human. Furthermore, we observe hyperrealism effects for some specific pairs of human voices and their corresponding voice clones (Fig 5). Even without finding evidence for a hyperrealism effect *per se* for voice clones, as it has been reported in analogous studies in the face perception literature, it is worth considering on a theoretical and conceptual level what it means that some real and natural human voices can sound less real than some AI-generated voices [33].

The voice clones used in our study, which were more effective in yielding perceptions of realness and humanness in the listeners, exemplified the state-of-the-art in low-demand, low-cost voice cloning. Thus, we demonstrate how voices generated from limited amounts of input data can reach a similar level of human likeness to real recordings of human speakers (see also Barrington et al. 2025) [20]. Of course, creating highly realistic recordings using AI-generated voices still requires some human intervention, via, e.g., screening and basic editing of audio files as was done in the current study. Similarly, it is not the case that every tool for AI-based audio generation will yield recordings of voices that sound as real as human voices. Finally, there may be use cases (be they benign or malicious) for which it is either not yet possible or simply not intended to create convincing AI-generated voices.

Nonetheless, at this point the widespread availability of humanlike AI-generated voices as created by commercially available products has numerous implications for how humans evaluate the voices they hear around them. Research on human voice perception has for example shown that hearing a voice humanises the speaker from the perspective of the listener – hearing a person speaking (versus reading a transcript of their words) has positive impacts on perceived employability [42], while also mitigating against the dehumanisation of opponents in the context of disagreement [43]. The Computers Are Social Actors theory [44] proposes that humans tend to unthinkingly treat computers as people, and recent supporting evidence suggests significant contributions of “social cues and constructs, attitudes, and behavio[u]rs” in human interactions with voiced artificial agents (Seaborn et al., 2021 [45], p.29). The finding that voice clones are indistinguishable from human voices therefore suggests that AI-generated voices are now apt to generate similar effects on listeners. Abercrombie, Cercas Curry, Dinkar and colleagues (2023) [46] highlight that there are important considerations for situations in which humanlike AI-generated voices are combined with other forms of AI, for example in the context of conversational agents, chatbots, and voice assistants. On the one hand, human users may experience frustration if an artificial agent that sounds convincingly humanlike does not exhibit compatible humanlike behaviours [47]. On the other, there is a risk that human users will engage in behaviours such as over-sharing or over-trusting due to the misattribution of human emotional and empathetic qualities to non-human machines. Relatedly, work has already investigated the extent to which anthropomorphised machines offer a dangerous test bed for inappropriate social behaviours, for example the sexual harassment of conversational agents that have been assigned ostensibly feminine personae [48,49].

In our experiments, we invited participants to evaluate human recordings and AI-generated stimuli for first impressions of trustworthiness and dominance. We found that both generic AI-generated voices and voice clones were perceived as overall more dominant than real human recordings, while the generic AI-generated voices in Experiment 1a sounded more trustworthy than human voices. In line with previous research [23], our findings suggest that it is possible for AI-generated speech to connote certain humanlike traits more strongly than human voices themselves, even when the generic AI-generated voices are not perceived as high in realness or humanness (Experiment 1a). Combining this observation with evidence from prior research on how first impressions of human voices can impact listener behaviours, including monetary investments [50] and voting choices [51,52], we suggest that AI voice design should be able to incorporate trade-offs between perceived humanness and perceived humanlike qualities. This could be important for maintaining human awareness of the artificiality of computer agents without necessarily sacrificing the desirability of the artificial interlocutor’s perceived qualities [53,54]. Future work in this area should explore the interplay of realness/humanness and trait impressions on humans’ reactions to voiced human and non-human agents.

Our findings do not necessarily reflect the ceiling of human listeners’ abilities to recognise AI-generated voices, nor the floor. The experiments reported here used recordings and clones of human voice identities that were unfamiliar to the participants, as well as pseudorandomly-generated generic AI voices with no specific corresponding human identity. Therefore, to perform the experimental tasks, the listeners were evaluating the voices in relation to their general representations of realness and humanness [33]. However, when considering voice cloning in particular, the motivation for many use cases is to generate a convincing likeness of a specific, familiar human identity. Experimental research into human recognition of familiar voices has shown that while this can be error-prone and vulnerable for weakly-known identities, perceptual representations of personally familiar others (e.g., romantic partners, friends) are likely to be very well-formed and robust [55]. Thus, it is possible that while state-of-the-art voice cloning may leave unfamiliar listeners unable to reliably detect a voice clone, Rosi et al. (2025) [23] the very same voice clone may not be as convincing to listeners with in-depth knowledge of the original speaker’s voice. Similarly, listeners with particular expertise in the area of audio engineering or auditory perception (e.g., musicians, phoneticians), or increased day-to-day exposure to AI-generated speech, may have enhanced sensitivities to AI compared with the general population (see previous findings for positive effects of AI and internet use experience on visual deep fake detection accuracy; Hulzebosch et al., 2020; Pehlivanoglu et al., 2024 [25,56]). Demographic factors may also influence listeners’ perceptions and biases: recent work found that older adults showed reduced sensitivity to AI-generated speech, and found it to sound more natural, compared with younger adult listeners, despite no group differences in self-reported experience with AI-generated speech [57]. Future work should address how individual differences in human listeners impact the evaluation of realness and humanness from AI-generated voices, and outline which factors drive these individual differences.

It is unclear whether further advances in generative voice AI will eventually achieve a level of realness that consistently exceeds that of human voice recordings. Since hyperrealistic AI-generated images of faces exist, it is possible that technology for AI-assisted voice generation will eventually also create hyperrealistic voices. However, one crucial difference between AI-generated images of faces and AI-generated voices is that face images are static while voice recordings are dynamic, with temporal changes encoding both linguistic and paralinguistic information. Humans are expert in perceiving and analysing this temporally-unfolding speech-related information from voices. As a result, it might be arguably a harder task to convince the human perceptual system that dynamic speech and voice information are hyperrealistic. Indeed, recent evidence on deep fake video perception has found that humans can detect deep fake videos with high accuracy that meets or outperforms that of machines [56,58], suggesting that humans have specialised capabilities for assessing the authenticity of dynamic visual information.

It is also worthwhile noting that while face and voice perception share many similarities, there are some differences in how this person-related information from these two modalities is processed [59]. For example, identity and perhaps also other person-related information is more accurately and robustly recognised and perceived from faces compared to voices [59–61]. Perhaps this processing difference could underpin the lack of a hyperrealism effect for voices, where there is no scope to identify something that is distinctly more real than human in the context of already imperfectly resolved perceptual representations of person-related information from voices.

Finally, we observed substantial context effects, where judgements of the human voice recordings that we used to determine our benchmark for human-like realness shifted depending on the nature of the AI-generated stimuli included in the task. It is particularly noteworthy that, in line with the existing literature looking at AI detection in the context of human stimuli and very convincing AI-generated stimuli (see Miller et al., 2023; Barrington et al. 2025), we observed particularly high rates of listeners labelling recordings of real human voices as being AI-generated (i.e., only 62% and 72% of human voice recordings respectively were labelled as human in the experiments including voice clones, Experiment 1b and 2). It is therefore worth considering how we in future studies can best benchmark whether a voice or face is real – or even hyperreal – given that (hyper)realism is always a comparative judgement between two or more data points in this context. Should we perhaps explicitly set expectations for listeners with regard to what sounds more or less real/human before they start the study? Should studies always include “filler” stimuli that cover a wide range of realness/humanness to implicitly norm realness ratings? It may well be that the overall levels of perceived realness/humanness do not matter in many experimental studies, such that observing a relative difference between conditions of interest is sufficient. Our replication of the results of Experiment 1a and 1b may speak for the latter being the case. However, more applied studies that seek to understand listeners’ vulnerability to deception or misinformation via AI-generated voices should perhaps consider the “base rate” of AI-generated voices within their tasks, such that this approximates the prevalence of AI-generated voices and voice clones in everyday experience (e.g., [58]).

Technological advances, as well as societal needs and expectations, will continue to change how AI-generated voices are created. Both changes in AI-assisted voice generation and in listeners' experience will therefore continue to affect how AI-generated voices sound and are perceived. This calls for continued efforts to map how perceptions of realness interact with other aspects of voice perception and how the holistic percept of AI-generated voices in turn influences listeners’ behaviours and attitudes.

### Public significance statement

AI-generated faces can look more realistic and human than real faces. For voices, we find AI-generated voices do not sound 'hyperreal' yet. However, they do sound as real and human-like as human voices. Our results therefore highlight how advanced AI voice technology has already become.

## Supporting information

S1 File Effects of Accent. This document outlines an analysis to examine the effects of speaker accent (British English vs US English) on perceptual ratings.
(PDF)
