# Supplementary material for: Voice clones sound realistic but not (yet) hyperrealistic
Source: PLoS One. 2025 Sep 24;20(9):e0332692. doi: 10.1371/journal.pone.0332692 (PMC12459763; doi:10.1371/journal.pone.0332692)

Supplementary Materials

S1: Effects of Accent

All our listeners were from the UK while our voices spanned a number of different accent (18 American, 18 British, 2 Australian and 2 Indian voices).

We report below an exploratory analysis of how accent with accent (American English vs British English) affected binary Human or AI judgements and realness ratings as a factor. We have excluded Australian and Indian voices due to the very limited number of voices (N=2) in our study.

Data for Experiments 1a,1b, and 2 are plotted below. Yellow = Human voices, dark blue = Generic AI-generated voices, Turquoise = Voice Clones.

We ran mixed models predicting Realness ratings and "Human or AI-generated" judgements from the voice type (human, generic AI-generated, voice clone), accent (American English, British English) and their interaction as fixed effects. Random effects were the same as reported in the main manuscript.

Where we observe main effects of accent, UK participants generally consider voices with British English accents to be more real and more likely to be human.

We also find one interaction between accent and voice type, for realness ratings in Experiment 1a. This interaction can be best explained by the presence of a ceiling effect for "Human voices" that have a British English accent. This ceiling effect reduces the difference between Human American and British English voices, while there is not such ceiling (or floor) effect for AI-generated voices. The interaction is therefore most likely an artefact of the measurement scale as opposed to being an interaction showing cognitively meaningful interactions between accent and our fixed effect, voice type.

Human or AI-generated: Experiment 1a

- Main effect of accent (OR = 0.09, CI = 0.07-0.13; $p < .001$)
- Main effect of voice type (OR = 1.82, CI = 1.31-2.53; $p < .001$)
- No interaction (OR = 1.28, CI = 0.82-2.00; $p = 0.277$)

Human or AI-generated: Experiment 1b

- No main effect of accent (OR = 0.74, CI = 0.49-1.12; $p = .159$)
- No main effect of voice type (OR = 1.45, CI = 0.96-2.20; $p = .075$)
- No interaction (OR = 1.27, CI = 0.71-2.27; $p = 0.424$)

Human or AI-generated: Experiment 2

- Main effect of accent (OR = 1.56, CI = 1.11-2.18; $p = .010$)
- Effect of AI-generated voice < Human voices (OR = 0.14, CI = 0.10-0.20; $p < 0.001$)
- No Effect of Voice Clone > Human voices (OR = 0.89, CI = 0.62-1.20; $p = 0.389$)
- No interaction (all ps for pairwise comparisons of levels > 0.05)

Realness: Experiment 1a

- Main effect of accent (OR = 7.59, CI = 4.37-10.80; $p < .001$)
- Main effect of voice type (OR = -24.64, CI = -26.85- -20.42; $p < .001$)
- **Interaction (**OR = 5.55, CI = 1.00-10.10; **p = 0.017)**

Realness: Experiment 1b

- No main effect of accent (OR = 4.48, CI = -0.17-9.09; $p = .057$)
- No main effect of voice type (OR = -1.86, CI = -6.48-2.75; $p = .429$)
- No interaction (OR = 3.43, CI = -3.10- 9.95; $p = 0.303$)

Realness Experiment 2

- Main effect of accent (OR = 7.09, CI = 2.70–11.48; p = .002)
- Effect of AI-generated voice < Human voices (OR = -21.10, CI = -25.49- - 16.71; p < 0.001)
- No Effect of Voice Clone > Human voices (OR = -1.37, CI = -5.76-3.02; p = 0.542)
- No interaction (all ps for pairwise comparisons of levels > 0.05)

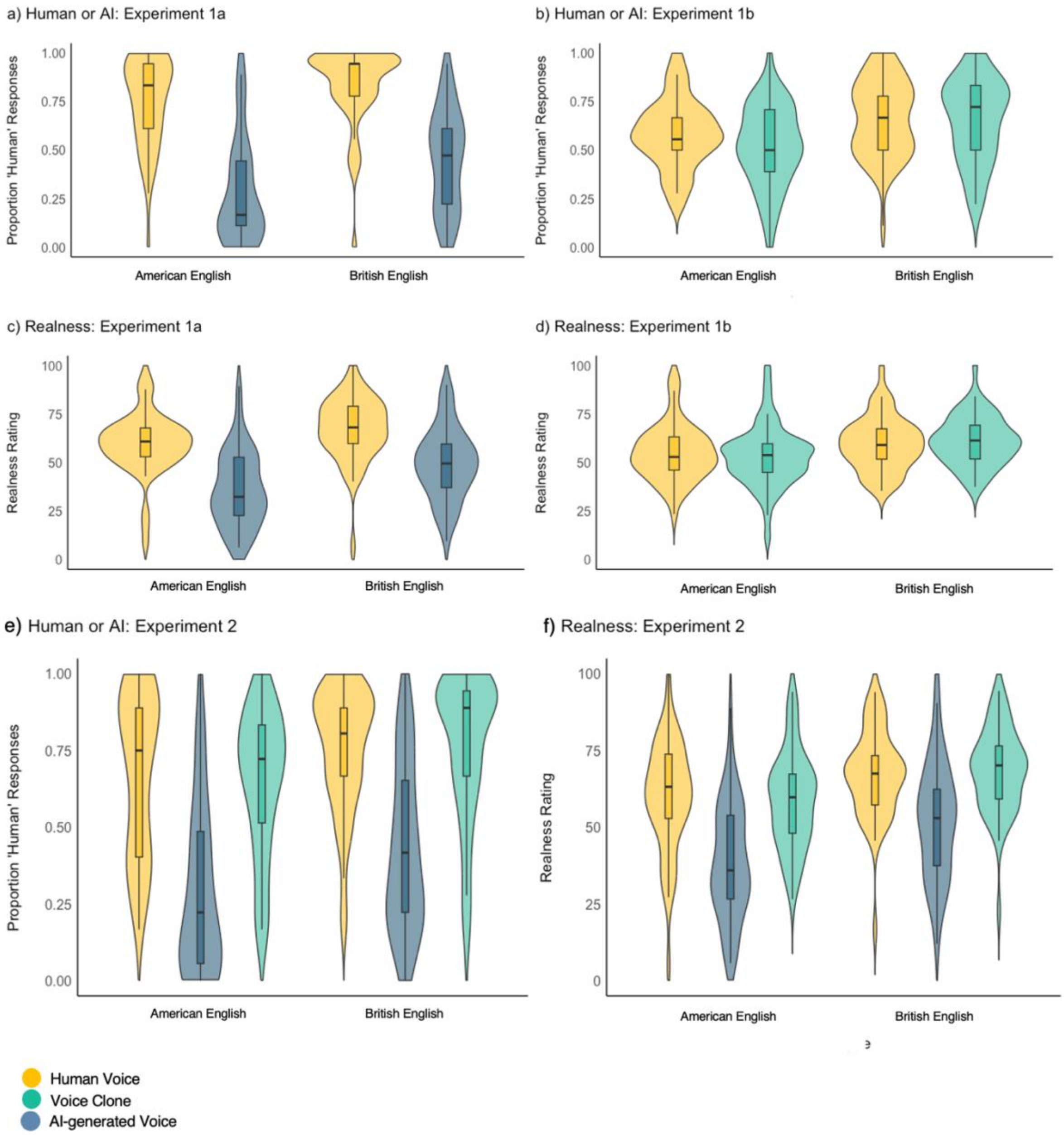

Supplement: S1 File — This document outlines an analysis to examine the effects of speaker accent (British English vs US English) on perceptual ratings. (PDF) [file pone.0332692.s001.pdf]
